# Closed-Incision Prophylactic Negative Pressure Wound Therapy in Emergency Laparotomy: A Systematic Review and Meta-Analysis of Randomized Controlled Trials

**DOI:** 10.7759/cureus.115652

**Published:** 2026-09-02

**Authors:** Saad A Alajmi, Ali K Alhilal, Abdullah Hussain

**Affiliations:** 1 Medicine and Surgery, Kuwait Institute for Medical Specializations, Kuwait City, KWT; 2 Nuclear Medicine, Kuwait Institute for Medical Specializations, Kuwait, KWT; 3 Medicine and Surgery, Kuwait Institute for Medical Specializations, Kuwait, KWT

**Keywords:** acute abdominal surgery, closed-incision negative pressure wound therapy, meta-analysis, negative-pressure wound therapy (npwt), postoperative complications, surgical site infection(ssi), systematic review (syst. rev.), emergency laparotomy

## Abstract

Surgical site infection (SSI) is common after emergency laparotomy, and closed-incision prophylactic negative pressure wound therapy (ciNPWT) applied over the closed incision has been proposed to reduce it, though trials disagree. We searched MEDLINE, Embase, CENTRAL, Scopus, and two trial registries to 21 June 2026, with no date or language restriction, for randomized controlled trials (RCTs) comparing ciNPWT with standard dressings in adults undergoing emergency laparotomy. The primary outcome was any incisional SSI within 30 days.

We used random-effects meta-analysis with restricted maximum likelihood, Hartung-Knapp-Sidik-Jonkman confidence intervals, and prediction intervals, together with RoB 2 and GRADE; because heterogeneity tracked study quality, we present the primary outcome stratified by risk of bias. Seven RCTs were included (1,351 patients; largest approximately 840), of which five contributed to the primary analysis and two to sensitivity analyses only - one because it reported outcomes only for a mixed elective and emergency whole cohort rather than an extractable emergency subgroup, and one because it was at high risk of bias with an implausibly high control-arm infection rate.

The single trial at low risk of bias showed no clinically important effect on incisional SSI [risk ratio (RR) 1.04, 95% confidence interval (CI) 0.83-1.30], whereas four small trials with some concerns showed a homogeneous reduction (RR 0.41, 0.28-0.59; I²=0%). The overall estimate was heterogeneous and non-significant (RR 0.54, 0.27-1.06; I²=67%; 95% prediction interval 0.09-3.02) and, being dominated by the single large trial, is not interpreted as a single effect; in leave-one-out analysis it became significant only when that trial was removed. Length of hospital stay (ratio of means 0.96, 0.86-1.07) and 30-day mortality (RR 0.95, 0.46-1.96) showed no difference. Among secondary outcomes, seroma was lower with ciNPWT (RR 0.59) and fascial dehiscence was uninformative (RR 1.15); the apparent SSI reduction was confined to the more subjectively assessed superficial component (RR 0.49), with no effect on deep SSI (RR 1.01). Certainty was low for the low-risk estimate (no benefit) and very low for the some-concern estimate (apparent benefit).

Current evidence does not provide even low-certainty support that ciNPWT reduces SSI after emergency laparotomy; adequately powered trials with masked outcome assessment are needed.

## Introduction and background

Emergency laparotomy is common in acute care surgery and carries high rates of postoperative wound complications. Surgical site infection (SSI), wound dehiscence, and related wound failures are frequent and drive morbidity, prolonged hospital stay, reoperation, and cost [1,2]. Patients undergoing emergency laparotomy are at particularly high risk because of intra-abdominal contamination, physiological derangement, and limited opportunity for preoperative optimization [3].

The consequences are not confined to the early postoperative period. Early wound failure is a recognized contributor to incisional hernia, which develops in a substantial minority of patients and in up to 40-50% of high-risk cases [4]. When primary fascial and skin closure is achieved, management of the closed incision is a distinct clinical decision, separate from open-abdomen and peritoneal negative pressure strategies [5]. Prophylactic negative pressure wound therapy applied over the closed incision (ciNPWT) is intended to reduce wound complications by lowering lateral wound tension, removing fluid, and improving perfusion [6].

Randomized trials of ciNPWT span many surgical settings, and several reviews report lower SSI rates, but these were conducted predominantly in elective or mixed surgical populations [6,7]. Evidence specific to emergency laparotomy is more limited and inconsistent. The largest and most rigorously conducted trial found no benefit, whereas several small single-centre trials reported large reductions. Existing syntheses have generally reported a single pooled benefit. However, a pooled estimate can be driven by smaller, lower-quality trials, so the direction and certainty of effect in the emergency setting remain unclear.

We therefore conducted a systematic review and meta-analysis of randomized controlled trials limited to emergency laparotomy, with a focus on risk of bias and heterogeneity. We aimed to determine whether ciNPWT reduces incisional SSI and other wound outcomes, and to grade the certainty of the evidence.

## Review

Methods

We report per PRISMA 2020 [8] and the Cochrane Handbook [9]; search reporting follows PRISMA-S [10]. The review was not registered, and no protocol was published in advance. Analytic decisions made after examining the data are identified as post-hoc and interpreted as exploratory (below).

Eligibility Criteria

We included randomized controlled trials enrolling adults undergoing emergency laparotomy, defined as a non-elective (urgent or emergent) laparotomy performed for an acute abdominal condition, with primary fascial and skin closure. Individual trials’ definitions of emergency laparotomy varied, and we applied each trial’s own definition. The intervention was prophylactic closed-incision negative pressure wound therapy applied to the closed incision immediately after skin closure. The comparator was any standard postoperative dressing without negative pressure, such as sterile gauze or an adhesive island dressing, with no restriction on the type of dressing. Trials had to report at least one wound-related outcome with a minimum follow-up of 30 days. No language or date restriction was applied.

We excluded non-randomized studies and reports available only as conference abstracts, editorials, case reports, or reviews. We also excluded studies of negative pressure therapy applied to open abdomens, delayed-closure wounds, or existing (non-prophylactic) wounds, and trials without a standard dressing comparator (that is, a dressing without negative pressure). Trials enrolling both elective and emergency patients were eligible for the primary analysis only when data specific to the emergency subgroup could be extracted; otherwise, their whole-cohort data were used in sensitivity analyses only.

Data Sources and Search Strategy

We undertook a structured search to identify randomized controlled trials of prophylactic closed-incision negative pressure wound therapy after emergency laparotomy. We searched MEDLINE (via PubMed), Embase, Scopus, and the Cochrane Central Register of Controlled Trials (CENTRAL) on 21 June 2026, with no date or language restriction. To reduce the risk of publication bias, we also searched the World Health Organization International Clinical Trials Registry Platform and ClinicalTrials.gov for unpublished, ongoing, or recently completed studies, and we screened the reference lists of included trials and relevant reviews. The complete search strategy for each source is provided in Appendix 1.

Study Selection

After removing duplicates with automated screening software (Rayyan), two reviewers independently screened titles and abstracts in duplicate to identify potentially eligible studies. Full-text articles were then assessed independently by the same two reviewers to confirm eligibility against the predefined criteria. Disagreements at either stage were resolved by discussion, with a third reviewer consulted when consensus could not be reached.

Data Extraction

Two reviewers independently extracted data using a standardized extraction form prepared before study selection, with discrepancies resolved by discussion and arbitration by a third reviewer when needed. Extracted items included bibliographic and methodological characteristics (authors, year, country, study design, and sample size); participant data (numbers allocated to each arm, age, sex, relevant comorbidities, and the indication for laparotomy); intervention details (device, applied pressure, duration of therapy, and timing relative to skin closure); the type of comparator dressing; and outcome data. All extracted values were checked against the source publications, and corresponding authors were contacted where information was missing or ambiguous. For one trial that reported only whole-cohort (mixed elective and emergency) data, we contacted the corresponding author to request the emergency-laparotomy subgroup; no reply was received, and that trial’s whole-cohort data were used in sensitivity analyses only.

Risk of Bias Assessment

Two reviewers independently assessed each trial with the Cochrane Risk of Bias 2 (RoB 2) tool [11], evaluating the randomization process, deviations from intended interventions, missing outcome data, measurement of the outcome, and selection of the reported result. Each domain and the overall judgement were rated as low risk, some concerns, or high risk, with disagreements resolved by discussion. Assessment targeted the effect of assignment to intervention, with particular attention to outcome-assessor masking for the subjectively assessed SSI outcome.

Outcome Measures

The primary outcome was any incisional SSI within 30 days (superficial and/or deep, defined by Centers for Disease Control and Prevention criteria; organ/space infection excluded). Secondary outcomes were superficial and deep incisional SSI, reported by depth, length of hospital stay, 30-day mortality, fascial (burst) dehiscence, superficial (skin) dehiscence analysed separately, and seroma; other outcomes were summarized narratively.

Statistical Analysis

We used the risk ratio (RR) for binary outcomes, chosen over the odds ratio because SSI is common and the RR is easier to interpret. For length of stay, we used the ratio of means on the log scale, converting medians to means by the method of Wan and colleagues [12], with a mean-difference sensitivity analysis. Random-effects models used restricted maximum likelihood with Hartung-Knapp-Sidik-Jonkman intervals [13]. To describe dispersion, we reported Q, I², τ², and a 95% prediction interval (t-distribution, k−2 df; ≥3 trials) [14]. Because prediction intervals are unstable when few trials are available, we interpreted them descriptively and did not give them precedence over the confidence interval. Multi-arm trials contributed a single combined intervention arm. Sensitivity and subgroup analyses included risk-of-bias subgroups, leave-one-out, a Baujat plot, an analysis excluding the largest trial, and an analysis limited to the trial at low risk of bias. Funnel plots and Egger’s test were not performed because fewer than 10 trials contributed to any outcome. Statistical significance was defined by a two-sided p<0.05, although we emphasized effect estimates with their confidence and prediction intervals over dichotomous significance testing. Analyses used R version 4.6.1 [15] with the metafor (version 5.0-1) [16] and meta (version 8.5-0) [17] packages, and certainty of evidence was rated with GRADE [18].

Handling of Heterogeneity

Heterogeneity in incisional SSI was concentrated along one dimension. The only trial at low risk of bias, which was multicentre and assessor-masked, showed no effect, whereas the small, mostly single-centre trials showed a homogeneous benefit. Risk of bias, trial size, and number of centres are mutually confounded and cannot be separated with five trials. We therefore present the SSI outcome stratified by risk of bias and treat the low-risk trial as the most credible estimate. The overall random-effects estimate is reported for reference only and is not interpreted as a single common effect, because averaging an unbiased null with bias-prone benefit estimates does not yield a clinically meaningful value. This presentation reflects study quality, not patient subgroups, since all trials enrolled emergency-laparotomy patients.

Post-hoc Analytic Decisions

Two analytic decisions were made after examining the data and are therefore post-hoc and exploratory: (A1) presenting the primary incisional-SSI outcome stratified by risk of bias and reporting the overall pooled estimate as a non-interpreted reference; and (A2) restricting one trial, Sahni et al. [24] to sensitivity analyses; this trial was at high risk of bias, and its implausibly high control-arm SSI rate corroborated that decision. Because the review was not registered, these should be read as data-driven rather than pre-specified.

Results

Search Results and Study Selection

Database searches returned 2,736 records (MEDLINE 458, Embase 1,083, CENTRAL 116, Scopus 1,079) and registers 81. After de-duplication, 1,430 unique database records and 68 register records were screened (1,498 in total). Screening excluded 1,404 records, and 94 full-text reports were assessed for eligibility, of which 87 were excluded, leaving 7 eligible trials. Registry screening identified no additional completed eligible trials. Two trials were restricted to sensitivity analyses, leaving five in the primary analysis: one, Di Re et al. [25], was a mixed elective/emergency cohort reported only at the whole-cohort level, and the other, Sahni et al. [24], had an implausibly high control-arm event rate in a single-centre, open-label trial. The study selection process is summarized in Figure 1.

**Figure 1:**
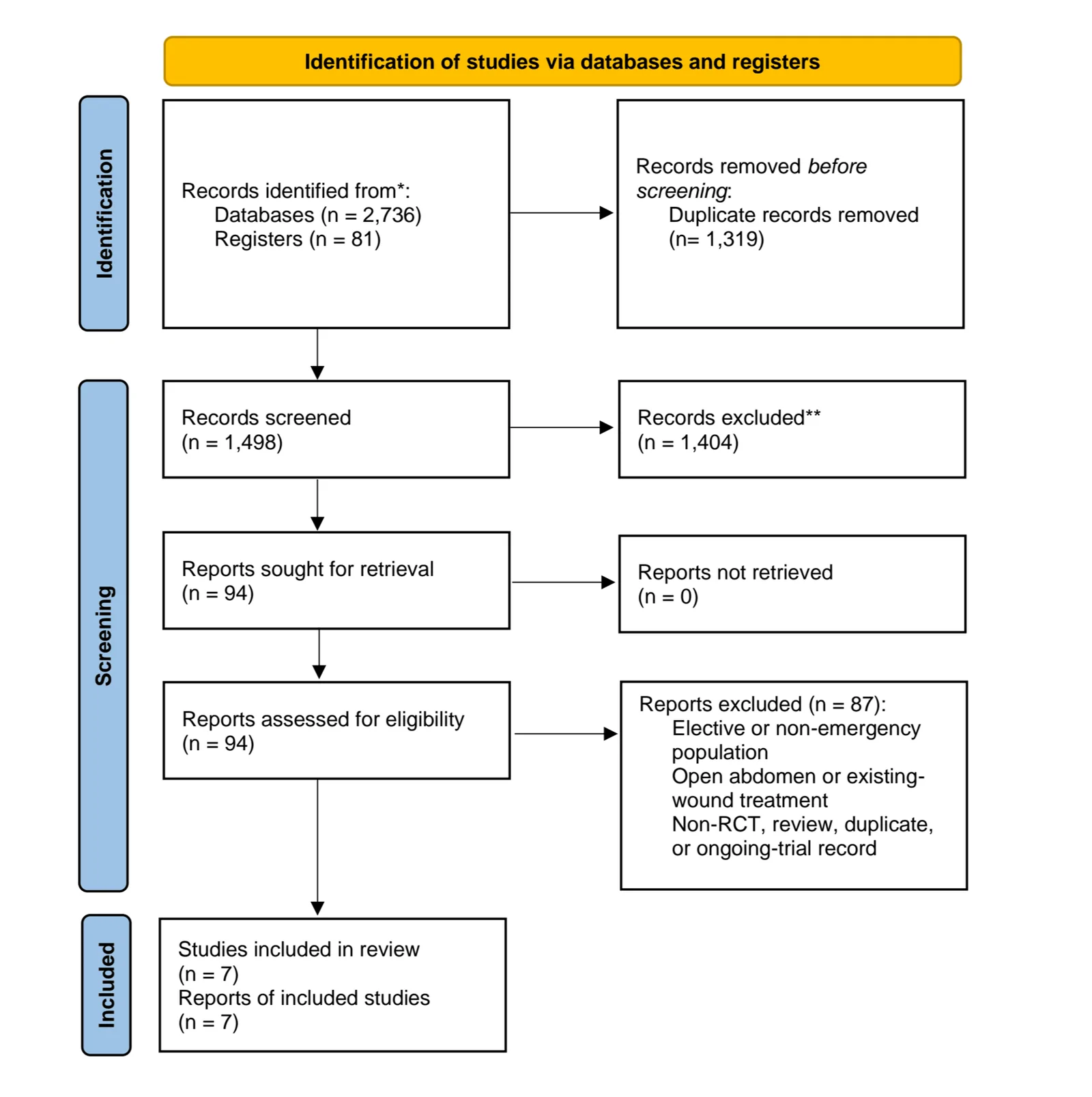
PRISMA flowchart outlining inclusion/exclusion of studies in the systematic review and meta-analysis [8]

Characteristics of Included Studies

The seven trials enrolled adults undergoing emergency laparotomy in the United Kingdom and Australia, Portugal, India, Malaysia, and Hungary; 1,351 patients contributed to the SSI comparisons (1,147 in the primary analysis). The largest trial randomized approximately 840 patients, of whom 788 were included in its SSI comparison after post-randomization exclusions and missing data. That trial was an assessor-masked, multicentre, pragmatic phase 3 RCT; the remaining trials were smaller and mostly single-centre, and most lacked blinded outcome assessment. Devices included single-use PICO and Prevena systems and vacuum-assisted closure, at −75 to −125 mmHg for three to seven days. Trial characteristics are summarized in Appendix 2, and baseline participant characteristics in Appendix 3.

Risk of Bias

Only the large multicentre trial Atherton et al. [19] was at low risk of bias in all five domains. Five trials raised some concerns overall, and one was at high risk; in several trials the concerns spanned more than one domain. Concerns about measurement of the outcome arose in the four trials in which masking of outcome assessment was absent or not described, given the partly subjective SSI endpoint: Costa et al. [20], Garg et al. [21], and Di Re et al. [25] raised some concerns, and Sahni et al. [24] was at high risk. The same four trials also raised concerns in the deviations-from-intended-intervention domain, because their reported analyses either did not include all randomized participants or did not specify the population analysed. Because the target estimand was the effect of assignment to intervention, the unavoidable lack of patient and surgeon masking was not in itself charged against this domain. Concerns in the randomization domain were confined to baseline imbalance in Herczeg et al. [23] and, in Sahni et al. [24], sealed envelopes that were not described as opaque or sequentially numbered. Missing outcome data raised concerns only in Philip et al. [22], whose complete-case analysis excluded a modest 11 to 16% of participants in broadly balanced proportions, and concerns about selection of the reported result arose only in Sahni et al. [24], for which no prospective registration or protocol was available. Sahni et al. [24] was the single trial at high risk overall, combining absent masking of a subjectively assessed outcome with an implausibly high control-arm event rate. Domain-level and summary judgements are shown in Figure 2.

**Figure 2:**
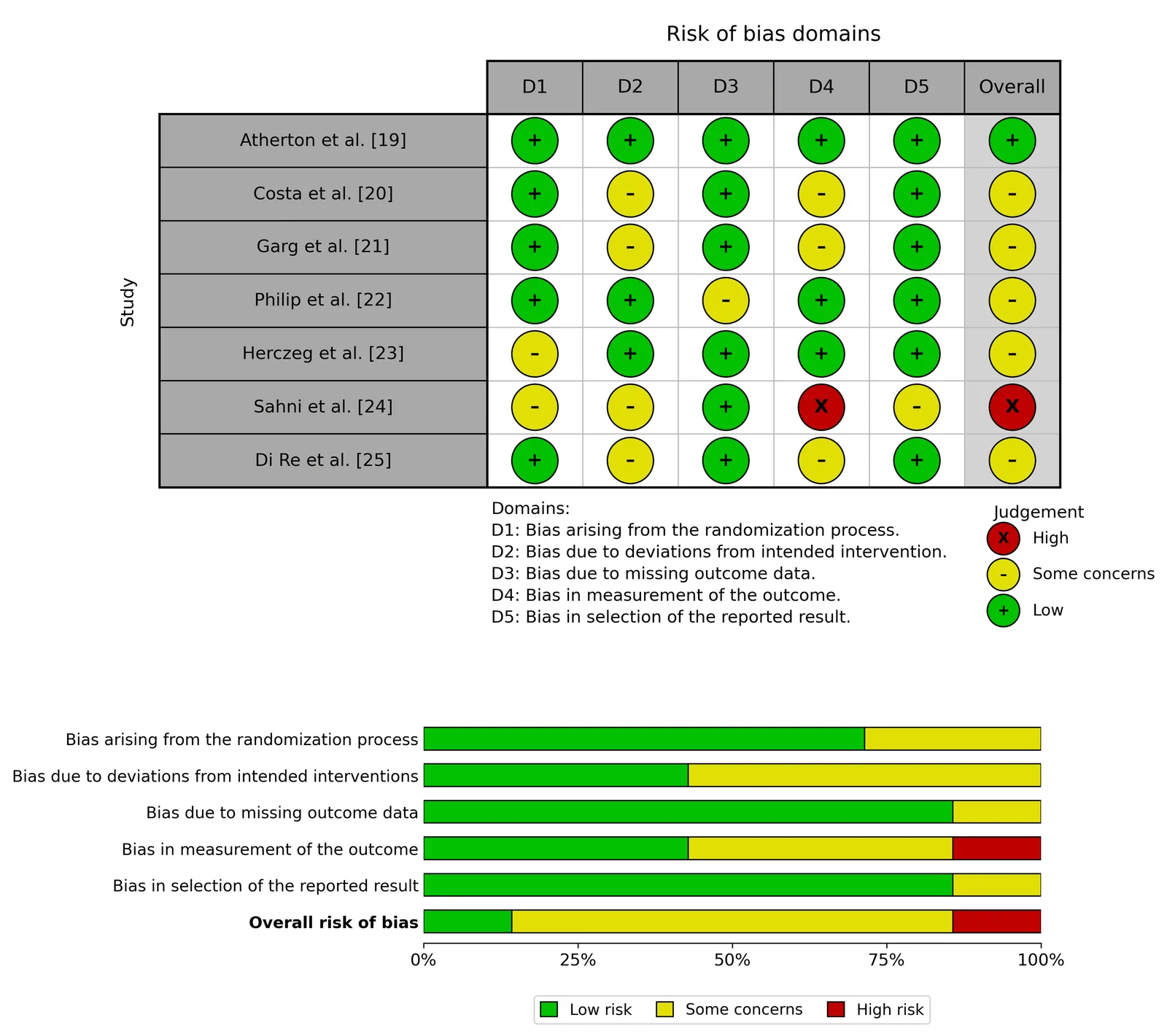
Risk of bias (RoB 2) of the included trials Risk of bias for the seven included trials, assessed with the Cochrane RoB 2 tool [11]. (A) Per-study traffic-light plot showing the judgement (low risk, some concerns, or high risk) for each of the five RoB 2 domains and overall; (B) distribution of judgements across studies. Trials assessed: [19-25].

Surgical Site Infection

The single trial at low risk of bias showed no evidence of a clinically important effect (RR 1.04, 95% CI 0.83-1.30; the interval is compatible with modest benefit or harm). The four small trials with some concerns about bias showed a homogeneous large reduction (RR 0.41, 0.28-0.59; I²=0%; Figure 3). The overall random-effects estimate was heterogeneous (RR 0.54, 0.27-1.06; I²=67%; τ²=0.24), with a 95% prediction interval of 0.09 to 3.02 that spans substantial benefit to substantial harm, and is reported for reference only. A test for subgroup difference (low risk vs some concerns) was significant (Q=13.8, df=1, p<0.001). Because risk of bias is confounded with trial size and, in most trials, single-centre design, this only localizes where the heterogeneity lies rather than identifying its cause, and it is regarded as exploratory

**Figure 3:**
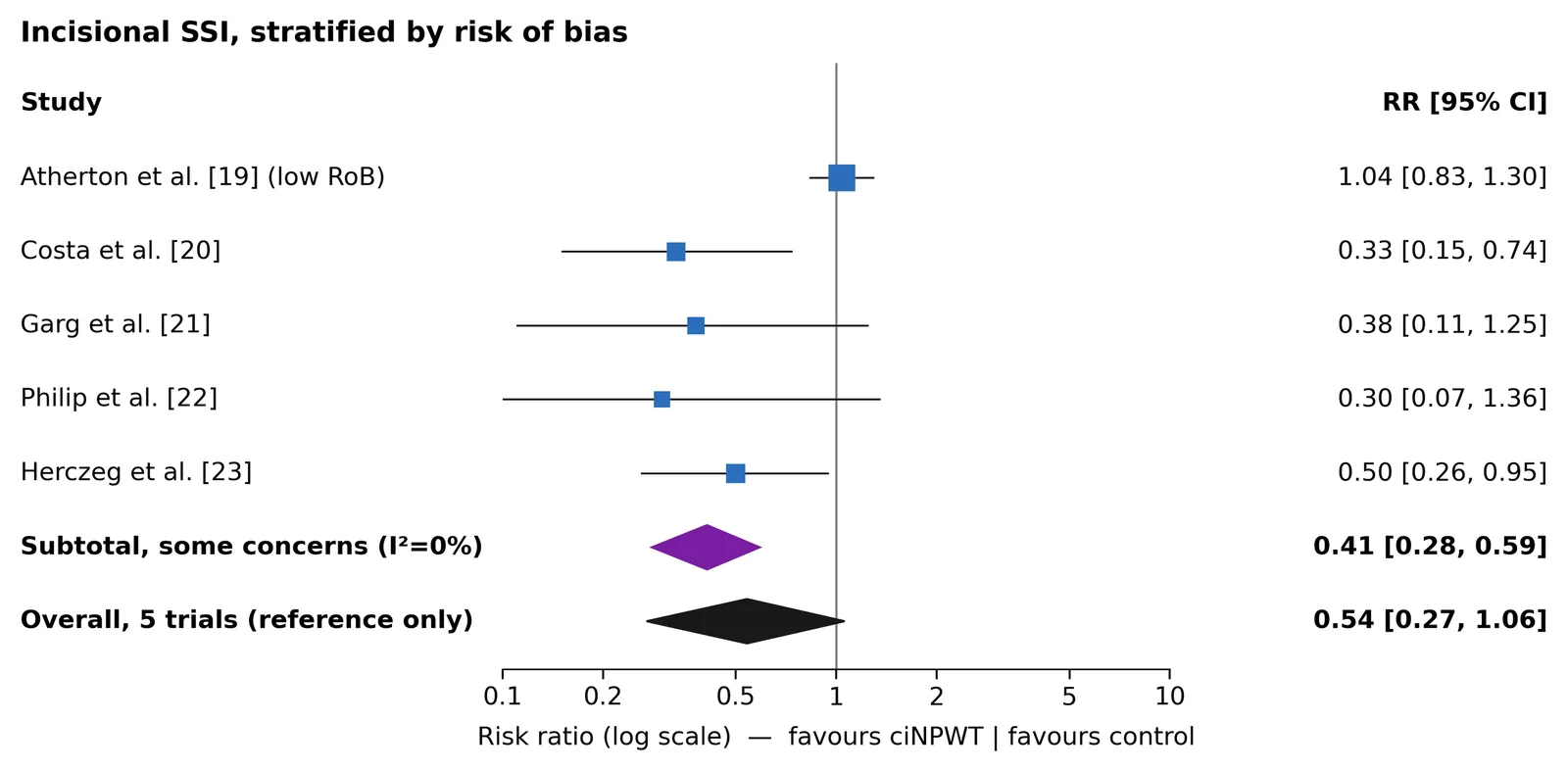
Risk-of-bias–stratified forest plot for incisional SSI Forest plot of incisional surgical site infection for the five primary-analysis trials [19-23], grouped by overall risk of bias. The single trial at low risk of bias, by Atherton et al. [19], is shown on its own (RR 1.04). "Subtotal, some concerns" is the pooled random-effects estimate of the four trials with some concerns about risk of bias [20-23]; RR 0.41, I²=0%. "Overall (reference only)" is the pooled estimate of all five primary trials (RR 0.54; I²=67%); it is shown for completeness but is not interpreted as a single effect because it is dominated by the one large low-risk trial and is highly heterogeneous. Squares are per-trial risk ratios (size proportional to weight); horizontal lines are 95% confidence intervals; diamonds are pooled estimates. RR, risk ratio; SSI, surgical site infection.

The homogeneity of the four smaller trials (I²=0%) is, in our interpretation, more consistent with small-study effects and shared methodological limitations than with a genuine common effect, although it is also compatible with a true homogeneous effect.

In sensitivity analyses that added the high-risk trial (RR 0.52, 0.31-0.87), the mixed-population whole cohort (RR 0.54, 0.32-0.92), or both (RR 0.53, 0.34-0.80), the confidence interval favoured ciNPWT and excluded the null, but every estimate remained substantially heterogeneous. The synthesis was governed by the single large trial at low risk of bias: in leave-one-out analysis the pooled estimate was non-significant whenever that trial was included and became significant (RR 0.41, 0.28-0.59; I²=0%) only when it was removed, whereas removing any smaller trial left the result essentially unchanged. Whether the synthesis appears to show benefit therefore depends entirely on the weight given to that one trial. A Baujat plot identified the largest trial as the principal contributor to heterogeneity (Figure 4), and the corresponding leave-one-out estimates are shown in (Figure 5).

**Figure 4:**
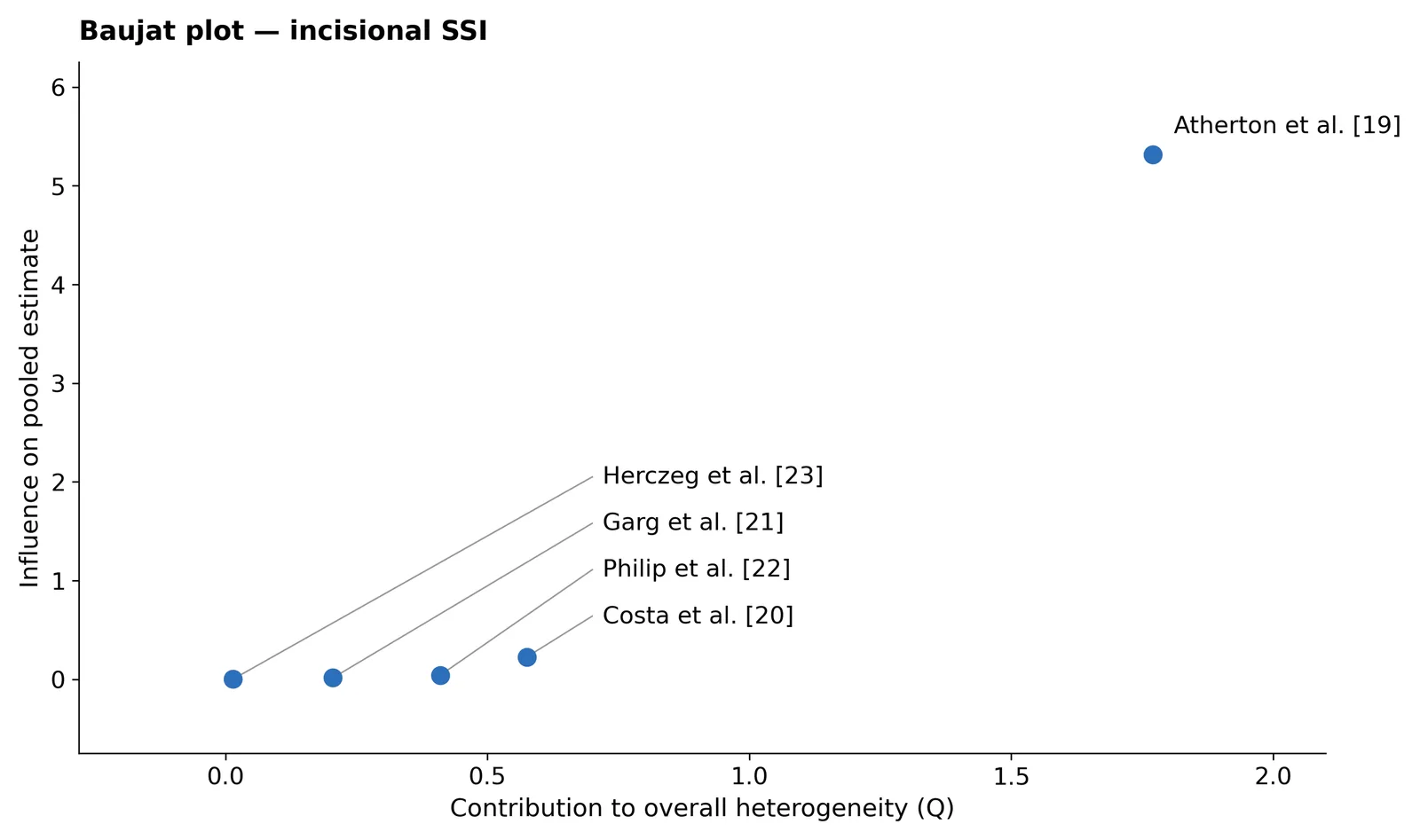
Baujat plot for incisional SSI Baujat plot for the incisional surgical site infection meta-analysis (five primary trials) [19-23]. The horizontal axis shows each trial’s contribution to the overall heterogeneity (Cochran’s Q); the vertical axis shows each trial’s influence on the pooled estimate. Trials farther to the right drive heterogeneity most, and those higher up most influence the pooled result. Atherton et al. [19], the single large low-risk-of-bias trial, is the dominant contributor on both axes; the four smaller trials cluster near the origin. SSI: surgical site infection.

**Figure 5:**
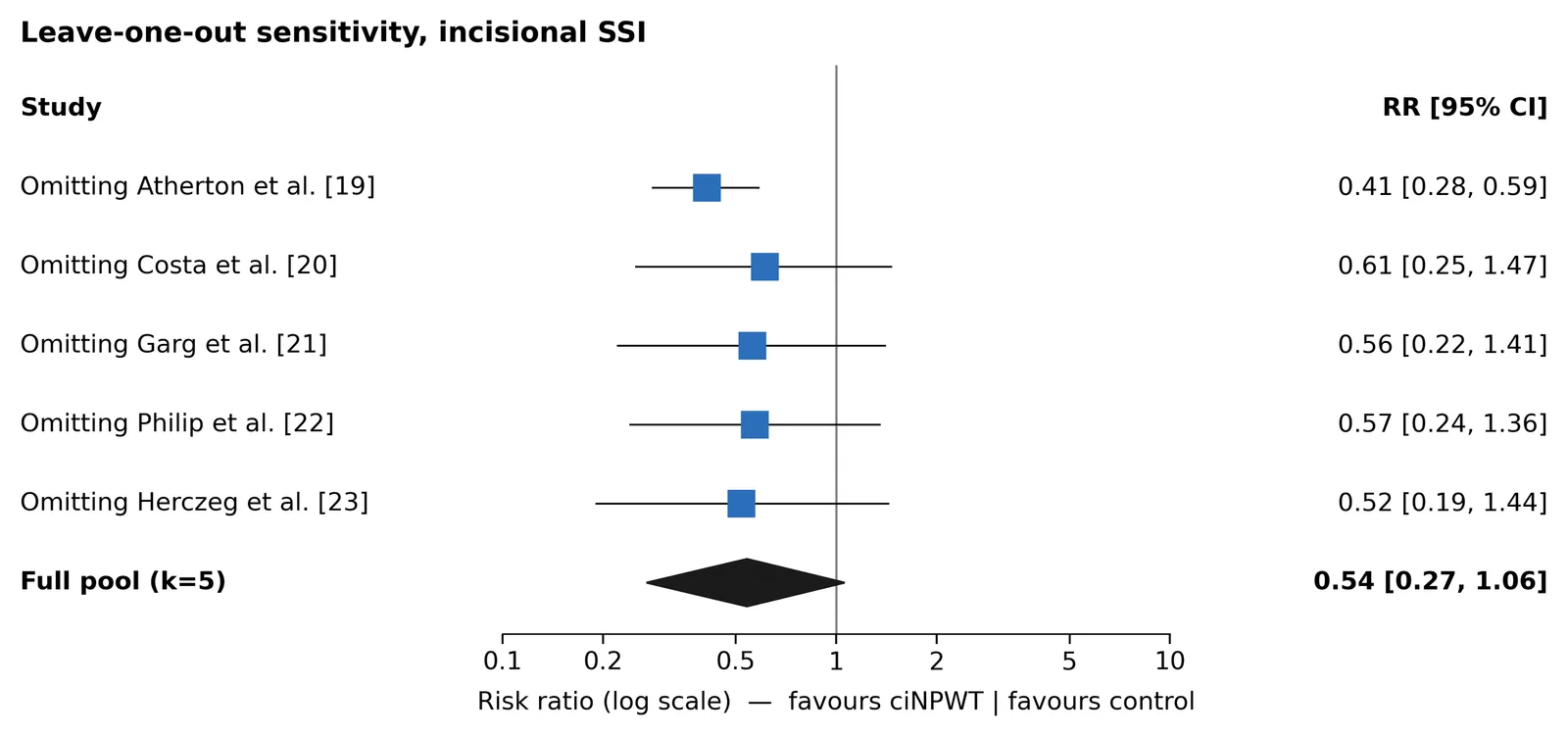
Leave-one-out sensitivity analysis for incisional SSI Leave-one-out sensitivity analysis for incisional surgical site infection, based on the five primary-analysis trials [19-23]. Each row shows the pooled random-effects risk ratio (and 95% confidence interval) recalculated after omitting the single named trial; the bottom row is the full five-trial pool (RR 0.54). This analysis interrogates the five primary trials only; the two sensitivity-only trials, Sahni et al. [24] and Di Re et al. [25], are not part of this pool. The pooled estimate remains non-significant when Atherton et al. [19] is included and becomes significant (RR 0.41, I²=0%) only when Atherton et al. [19] is removed, demonstrating that any apparent benefit depends entirely on the weight given to that one large trial. RR, risk ratio; SSI, surgical site infection.

Surgical Site Infection by Depth

Where reported by depth (two trials), superficial SSI favoured ciNPWT (RR 0.49, 0.37-0.66), whereas deep SSI showed no difference (RR 1.01, 0.83-1.22). This pattern rests on only two trials and is interpreted cautiously.

Length of Hospital Stay

Length of hospital stay showed no difference (ratio of means 0.96, 0.86-1.07; I²=0%; five trials), unchanged on the mean-difference sensitivity analysis and after adding the sensitivity-only trial (Figure 6).

**Figure 6:**
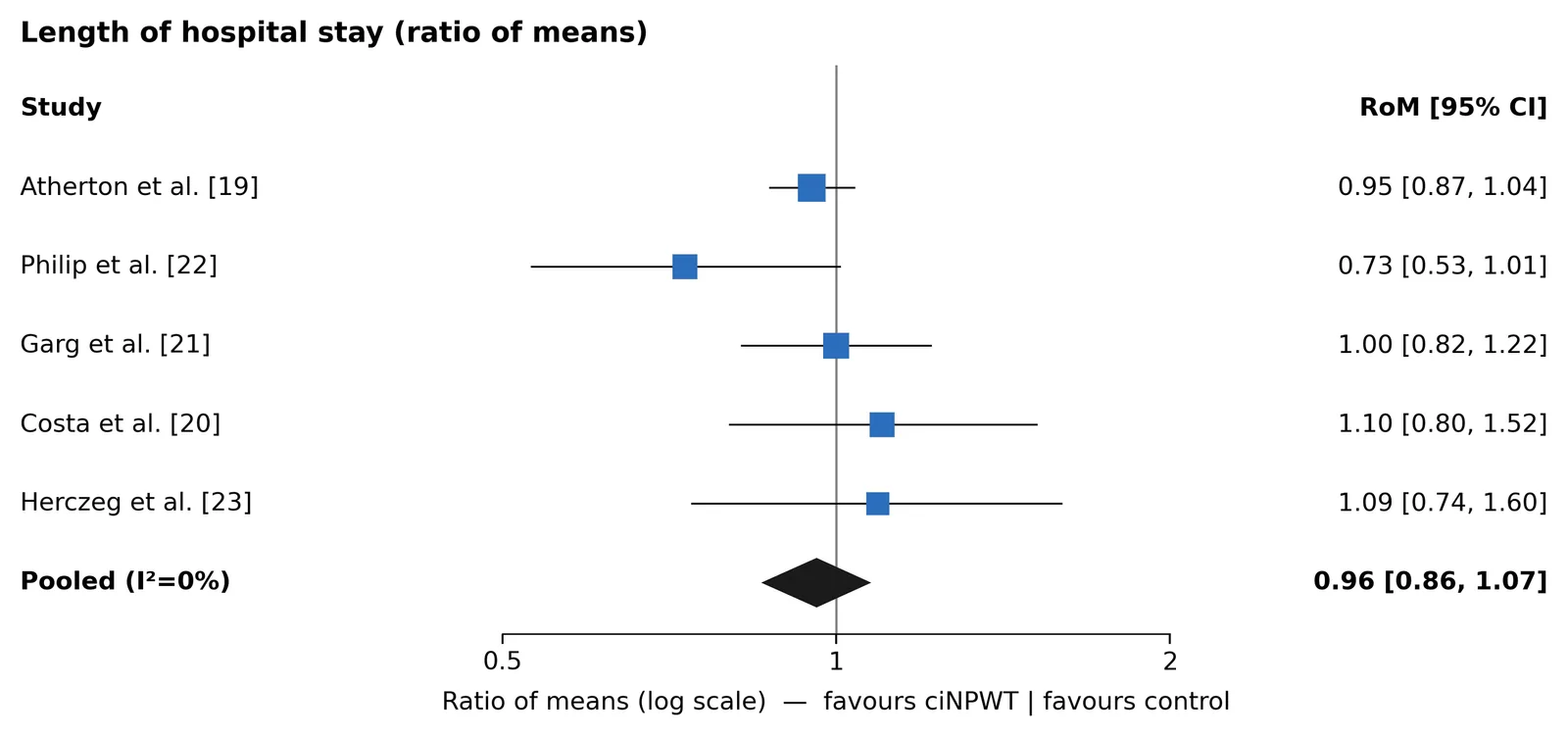
Length of hospital stay, ratio of means (log scale) Forest plot of length of hospital stay expressed as the ratio of means on the log scale, for the five primary-analysis trials [19-23]. A ratio of means below 1 favours ciNPWT. The pooled estimate showed no difference (ratio of means 0.96, 95% CI 0.86–1.07; I²=0%). ciNPWT, closed-incision prophylactic negative pressure wound therapy.

Wound Dehiscence

Fascial (burst) dehiscence was uninformative (RR 1.15, 0.19-7.14; I²=0%; three trials; very few events; Figure 7). Superficial (skin) dehiscence was reported by single trials only and is summarized narratively.

**Figure 7:**
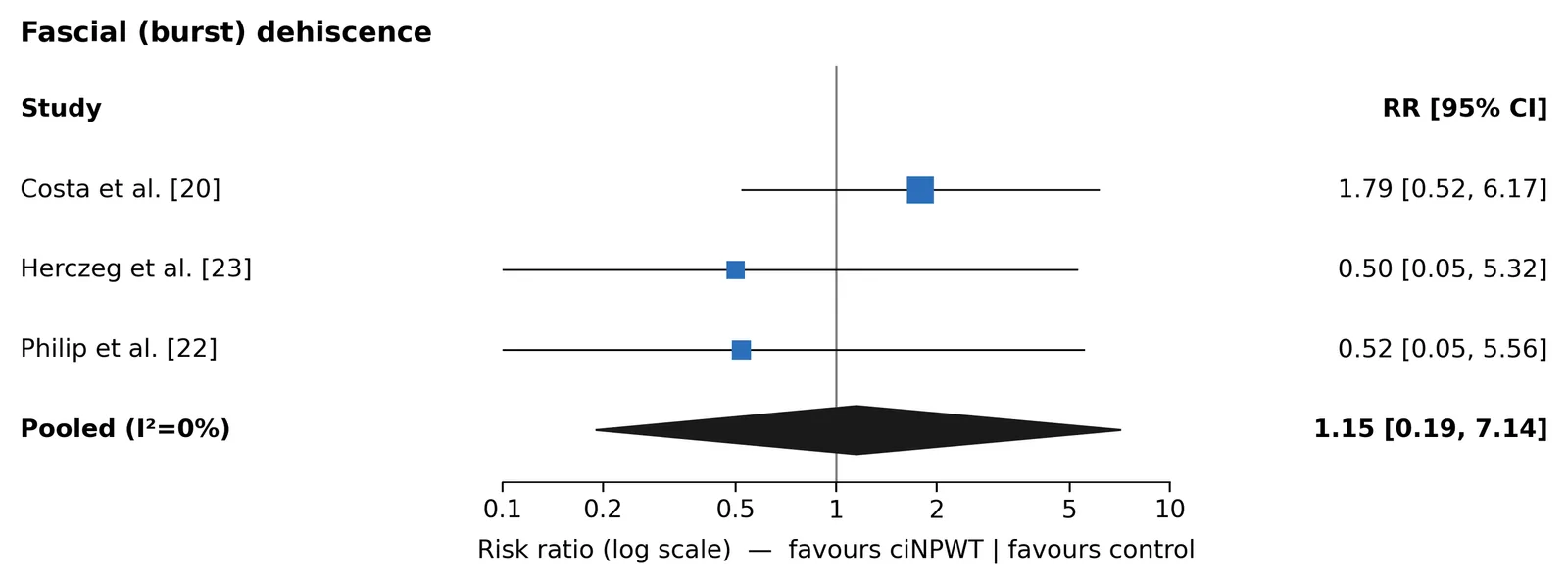
Fascial (burst) dehiscence forest plot Forest plot of fascial (burst) dehiscence for the three trials reporting this outcome [20,22,23]. The pooled estimate was uninformative owing to very few events (RR 1.15, 95% CI 0.19–7.14; I²=0%). RR, risk ratio.

Mortality

Thirty-day mortality showed no difference (RR 0.95, 0.46-1.96; I²=0%; four trials).

Seroma

Seroma was lower with ciNPWT (RR 0.59, 0.47-0.73; three trials). This estimate derives entirely from small trials with some concerns about bias and variable seroma definitions, and seroma is itself an ascertainment-sensitive outcome. It therefore does not provide independent confirmation of benefit (certainty very low).

Other Outcomes

Haematoma, reoperation, readmission, and composite complications were each reported by a single trial and are summarized narratively in Appendix 4.

Certainty of Evidence

In GRADE terms, certainty was low for the estimate from the low-risk trial (a single large trial showing no benefit) and very low for the estimate from the trials with some concerns (apparent benefit; downgraded for risk of bias and, more seriously, for small-study effects and non-replication in the low-risk trial). Certainty was moderate for length of stay and low for mortality (both null), and very low for seroma. A GRADE Summary of Findings is provided in Appendix 5.

Discussion

In this review restricted to emergency laparotomy, ciNPWT did not show a credible reduction in incisional SSI. The apparent benefit on unstratified pooling was confined to small, mostly single-centre trials and was absent in the only large trial, which was assessor-masked and at low risk of bias. Heterogeneity aligned with study quality rather than with clinical factors, and risk of bias, trial size, and number of centres cannot be separated with so few trials. We therefore anchored interpretation on the low-risk trial and treated the pooled estimate as a reference only. The prediction interval spans substantial benefit to substantial harm, indicating that the result of a future trial remains highly uncertain and could show no effect or harm.

Three observations support this cautious reading. First, the divergence is structured by trial size and quality: the benefit is concentrated in small, mostly single-centre trials that each carry concerns in one or more domains (absent or unreported masking of outcome assessment, analyses that did not include all randomized participants or did not specify the population analysed, complete-case analysis, or baseline imbalance), whereas the single large, multicentre trial, which had masked outcome assessment and low risk of bias, found no effect. Although small-study effects cannot be formally tested with fewer than ten trials, this pattern is the kind expected from such effects, and we treat it as suggestive rather than confirmed. Second, length of stay, the outcome least susceptible to ascertainment bias, showed no benefit; mortality, for which ciNPWT has no plausible mechanism, was likewise null. Third, within the SSI outcome, the reduction was confined to superficial SSI, the more subjectively assessed component (RR 0.49), while deep SSI was null (RR 1.01), a dissociation compatible with detection bias, although it rests on only two trials. The seroma reduction does not alter this interpretation, as it derives from the same small trials and is graded very low.

Our conclusions are more cautious than analyses that emphasize a single pooled estimate. Three choices explain this difference. We kept strict fidelity to the emergency-laparotomy population, we presented estimates stratified by risk of bias with prediction intervals rather than a single pooled effect, and we applied formal GRADE appraisal. Read this way, the most credible single estimate indicates no benefit at low certainty, while the apparent benefit in the smaller trials is of very low certainty.

Strengths and limitations

Strengths include a comprehensive multi-database search, fidelity to the emergency-only population, conservative HKSJ estimation with prediction intervals, analysis stratified by risk of bias, and GRADE appraisal. The main limitation is the evidence base itself. The trials are few and mostly small; they vary in device, pressure, and duration, and all but one carry some concerns or high risk of bias. With fewer than ten trials per outcome, small-study effects could not be assessed formally. The review was not registered; the primary framing stratified by risk of bias, and the restriction of trials to sensitivity analyses, were post-hoc, data-driven decisions and should be interpreted as exploratory. Web of Science was not searched, but given the substantial overlap with Scopus, the effect on yield is likely negligible.

Implications

Routine adoption of ciNPWT after emergency laparotomy is not supported by high-certainty evidence. Whether ciNPWT reduces SSI when the outcome is masked can be answered only by adequately powered trials with masked outcome assessment. Until then, the most reliable available estimate indicates no benefit.

## Conclusions

Current randomized evidence does not provide convincing support that closed-incision prophylactic negative pressure wound therapy reduces surgical site infection after emergency laparotomy. The apparent benefit seen on simple pooling is confined to small, mostly single-centre trials with methodological concerns and is not reproduced in the only large, rigorously conducted trial. Length of stay and mortality are unaffected. Prophylactic negative pressure therapy should therefore not be adopted routinely in this setting based on current evidence, and adequately powered trials with masked outcome assessment are needed to determine whether a real benefit exists.

## Appendices

Appendix 1 

**Table 1:** Search strategy (PRISMA-S)

Source (interface)	Search strategy as run	Date run	Records
MEDLINE (via PubMed)	("Negative-Pressure Wound Therapy"[tiab] OR "NPWT"[tiab] OR "topical negative pressure"[tiab] OR "incisional negative pressure"[tiab] OR "closed incision negative pressure"[tiab] OR "negative pressure dressings"[tiab] OR "vacuum assisted closure"[tiab] OR "Prevena"[tiab] OR "Negative-Pressure Wound Therapy"[MeSH]) AND ("Laparotomy"[tiab] OR "laparotomies"[tiab] OR "Laparotomy"[MeSH] OR "emergency abdominal surgery"[tiab] OR "midline incision"[tiab] OR "abdominal surgery"[tiab])	21 June 2026	458
Embase (Elsevier)	('negative pressure wound therapy'/exp OR 'negative pressure wound therapy':ti,ab OR 'npwt':ti,ab OR 'topical negative pressure':ti,ab OR 'incisional negative pressure':ti,ab OR 'closed incision negative pressure':ti,ab OR 'negative pressure dressing*':ti,ab OR 'vacuum assisted closure':ti,ab OR 'prevena':ti,ab OR 'pico dressing':ti,ab) AND ('laparotomy'/exp OR 'laparotomy':ti,ab OR 'laparotomies':ti,ab OR 'emergency abdominal surgery':ti,ab OR 'midline incision':ti,ab OR 'abdominal surgery':ti,ab)	21 June 2026	1,083
Cochrane CENTRAL (Cochrane Library)	("negative pressure wound therapy" OR NPWT OR "topical negative pressure" OR "incisional negative pressure" OR "closed incision negative pressure" OR "negative pressure dressing*" OR "vacuum assisted closure" OR Prevena OR "PICO dressing") AND (laparotomy OR laparotomies OR "emergency abdominal surgery" OR "midline incision" OR "abdominal surgery"); Title-Abstract-Keyword	21 June 2026	116
Scopus	TITLE-ABS-KEY("negative pressure wound therapy" OR NPWT OR "topical negative pressure" OR "incisional negative pressure" OR "closed incision negative pressure" OR "negative pressure dressing*" OR "vacuum assisted closure" OR Prevena OR "PICO dressing") AND TITLE-ABS-KEY(laparotomy OR laparotomies OR "emergency abdominal surgery" OR "midline incision" OR "abdominal surgery")	21 June 2026	1,079
WHO ICTRP (register)	Title: laparotomy OR abdominal OR peritonitis OR emergency surgery; Intervention: negative pressure OR NPWT OR vacuum assisted closure OR Prevena OR PICO; synonyms on; all recruitment statuses	21 June 2026	64
ClinicalTrials.gov (register)	Intervention: NPWT / Prevena / PICO / vacuum-assisted-closure terms; Condition: laparotomy or emergency or general abdominal surgery; study type: interventional (subset of ICTRP, de-duplicated against it)	21 June 2026	17
Reference lists of included trials and relevant reviews (hand-search)	Backward citation screening	21 June 2026	0 additional

Appendix 2 

**Table 2:** Characteristics of included trials [19-25]

Trial (year)	Country	Design / blinding	Population	Device / pressure / duration	NPWT / control (SSI)	RoB	Role
Atherton et al. [19]	UK & Australia	Pragmatic phase 3; assessor-masked; multicentre	Emergency laparotomy, incision ≥5 cm	PICO / −80 mmHg / 7 d	394 / 394 (randomized ≈840)	Low	Primary
Costa et al. [20]	Portugal	3-arm RCT; no blinding/concealment	Urgent laparotomy, mixed	Prevena & PICO / NR / 7 d	80 / 43 (arms combined)	Some concerns	Primary
Garg et al. [21]	India	RCT, computer-generated randomization; single-centre	Emergency midline laparotomy	VAC / −75 mmHg intermittent / 3 d	25 / 25	Some concerns	Primary
Philip et al. [22]	Malaysia	Double-blind RCT; single-centre	Emergency laparotomy, midline <35 cm	PICO / −80 mmHg / 7 d	47 / 49	Some concerns (complete-case)	Primary
Herczeg et al. [23]	Hungary	Multicentre RCT	Emergency laparotomy, high-risk wound	VAC + silver/PVA / −90 mmHg / 5 d	45 / 45	Some concerns	Primary
Sahni et al. [24]	India	RCT; single-centre; open-label	Emergency midline laparotomy, peritonitis	VAC / −75 mmHg / 4 d	40 / 40	High	Sensitivity
Di Re et al. [25]	Australia	Prospective; non-blinded; multicentre	Open abdominal, elective+emergency (49% emergency)	Prevena / −125 mmHg / 5–7 d	61 / 63 (whole cohort)	Some concerns	Sensitivity

Appendix 3 

**Table 3:** Baseline characteristics of participants [19-25]

Trial (year)	N (ciNPWT / control)	Age, years (ciNPWT / control)	Male, % (ciNPWT / control)	Population / indication
Atherton et al. [19]	394 / 394	63.8 (whole trial)	48 (whole trial)	Emergency laparotomy, midline incision ≥5 cm
Costa et al. [20]	80 / 43	63.4 / 64.1	72.5 / 62.8	Urgent or emergency laparotomy; clean-contaminated or contaminated
Garg et al.[21]	25 / 25	46.8 / 42.0	48.0 / 64.0	Emergency midline laparotomy
Philip et al. [22]	47 / 49	NR	55 / 55	Emergency laparotomy; clean-contaminated or dirty wounds
Herczeg et al. [23]	45 / 45	61.0 / 67.7	57.8 / 46.7	Emergency laparotomy, high-risk wound
Sahni et al. [24]	40 / 40	26.8 / 33.8	70.0 / 75.0	Emergency midline laparotomy for peritonitis
Di Re et al. [25]	61 / 63	70 / 66	49.2 / 57.1	Open abdominal surgery, elective and emergency (49% emergency)

Appendix 4 

**Table 4:** Secondary and narrative outcomes (single-trial estimates) [19, 20, 25]

Outcome	Trial	ciNPWT (n/N)	Control (n/N)	RR (95% CI)
Haematoma	Costa et al.	4/80	2/43	1.08 (0.21–5.63)
Reintervention	Costa et al.	19/80	13/43	0.79 (0.43–1.43)
Reoperation	Di Re et al. (whole cohort)	6/61	3/63	2.07 (0.54–7.89)
Wound-related readmission	Atherton et al.	11/399	11/398	1.00 (0.44–2.27)
Readmission (any cause)	Costa et al.	9/80	2/43	2.42 (0.55–10.70)
Any complication	Costa et al.	51/80	24/43	1.14 (0.84–1.56)
Wound complications excluding SSI	Atherton et al.	73/392	71/397	1.04 (0.77–1.40)
Complications (Clavien-Dindo ≥2)	Di Re et al. (whole cohort)	33/61	26/63	1.31 (0.90–1.91)

Appendix 5

**Table 5:** GRADE Summary of Findings

Outcome (studies)	Relative effect (95% CI)	Absolute effect (per 1000)	Certainty	Main reasons for certainty rating
Incisional SSI, low risk of bias (1 RCT, n=788)	RR 1.04 (0.83–1.30)	274 to 284; no difference	Low	Single-trial stratum (most credible)
Incisional SSI, some concerns (4 RCTs, n=359)	RR 0.41 (0.28–0.59), I²=0%	296 to 121; difference −175	Very low	Risk of bias (−1); small-study effects, not replicated in the low-risk trial (−2)
Overall pooled SSI, reference only, not interpreted (5 RCTs)	RR 0.54 (0.27–1.06); I²=67%; 95% PI 0.09–3.02	Not interpretable	Not applicable	Heterogeneity explained by study quality
Length of hospital stay (5 RCTs)	RoM 0.96 (0.86–1.07), I²=0%	Approximately no difference	Moderate	Some risk of bias; median-to-mean conversion
30-day mortality (4 RCTs)	RR 0.95 (0.46–1.96)	47 to 44; no difference	Low	Very serious imprecision
Fascial (burst) dehiscence (3 RCTs)	RR 1.15 (0.19–7.14)	Uninformative	Very low	Risk of bias; very serious imprecision
Seroma (3 RCTs)	RR 0.59 (0.47–0.73), I²=0%	222 to 131; difference −91	Very low	Risk of bias; small trials; ascertainment-sensitive outcome
